# Intracellular responses to frequency modulated tones in the dorsal cortex of the mouse inferior colliculus

**DOI:** 10.3389/fncir.2013.00007

**Published:** 2013-01-31

**Authors:** H.-Rüdiger A. P. Geis, J. Gerard G. Borst

**Affiliations:** Department of Neuroscience, Erasmus MC, University Medical Center RotterdamRotterdam, Netherlands

**Keywords:** patch-clamp, direction selectivity, rate selectivity, FM reconstruction, auditory midbrain

## Abstract

Frequency modulations occur in many natural sounds, including vocalizations. The neuronal response to frequency modulated (FM) stimuli has been studied extensively in different brain areas, with an emphasis on the auditory cortex and the central nucleus of the inferior colliculus. Here, we measured the responses to FM sweeps in whole-cell recordings from neurons in the dorsal cortex of the mouse inferior colliculus. Both up- and downward logarithmic FM sweeps were presented at two different speeds to both the ipsi- and the contralateral ear. Based on the number of action potentials that were fired, between 10 and 24% of cells were selective for rate or direction of the FM sweeps. A somewhat lower percentage of cells, 6–21%, showed selectivity based on EPSP size. To study the mechanisms underlying the generation of FM selectivity, we compared FM responses with responses to simple tones in the same cells. We found that if pairs of neurons responded in a similar way to simple tones, they generally also responded in a similar way to FM sweeps. Further evidence that FM selectivity can be generated within the dorsal cortex was obtained by reconstructing FM sweeps from the response to simple tones using three different models. In about half of the direction selective neurons the selectivity was generated by spectrally asymmetric synaptic inhibition. In addition, evidence for direction selectivity based on the timing of excitatory responses was also obtained in some cells. No clear evidence for the local generation of rate selectivity was obtained. We conclude that FM direction selectivity can be generated within the dorsal cortex of the mouse inferior colliculus by multiple mechanisms.

## Introduction

Most natural sounds vary in the frequency domain. Frequency modulation (FM) is an important component of animal communication, including speech (Stein, 1968; Ryan, 1983; Kanwal et al., 1994; Holy and Guo, 2005; Zeng et al., 2005). In addition, FM has been described as a component of echolocation calls in bats and other animals (Simmons and Stein, 1980; Siemers et al., 2009). Within the auditory system, many neurons respond to FM stimuli and a subset of those cells preferentially fire action potentials in response to FM sweeps with a certain rate or direction. These neurons are considered rate- or direction-selective. FM direction selective neurons have been found in the major nuclei of the primary ascending auditory pathway, including the cochlear nucleus, inferior colliculus, medial geniculate body, and the auditory cortex (Whitfield and Evans, 1965; Erulkar et al., 1968; Hage and Ehret, 2003; Lui and Mendelson, 2003; Zhang et al., 2003; Kuo and Wu, 2012). However, there is still relatively little FM direction selectivity in the cochlear nucleus, a major source of inputs to the inferior colliculus. As a result, the mechanisms generating FM selectivity in the central nucleus of the inferior colliculus have received a lot of attention. These studies have demonstrated that spectrally asymmetric synaptic inhibition plays an important role in creating FM direction selectivity within the inferior colliculus (reviewed in Fuzessery et al., 2011; Pollak et al., 2011). In these cells, for the preferred sweep direction excitation precedes inhibition, whereas for the other direction inhibition coincides with excitation. It is still debated whether this mechanism can entirely explain FM direction selectivity within the inferior colliculus, or whether there is an additional role for upstream processing or the timing of excitatory inputs (Suga, 1965; Clopton and Winfield, 1974; Poon et al., 1992; Felsheim and Ostwald, 1996; Gittelman et al., 2009; Williams and Fuzessery, 2011; Kuo and Wu, 2012).

Selectivity for the rate of FM sweeps can also be created by the spectrotemporal interaction of inhibitory and excitatory inputs, but additional mechanisms are thought to play a role as well (Gordon and O'Neill, 1998; Fuzessery et al., 2006; Williams and Fuzessery, 2011, 2012). Much of the rate selectivity of inferior colliculus neurons appears to be already present in their synaptic inputs (Williams and Fuzessery, 2010, 2011; Gittelman and Li, 2011).

Much less is known about FM direction and rate selectivity in the lateral and dorsal cortex than in the central nucleus of the inferior colliculus. The available evidence suggests that the lateral cortex contains a larger proportion of FM selective cells than the dorsal cortex (Poon et al., 1992; Gordon and O'Neill, 2000). The dorsal cortex of the inferior colliculus receives inputs from the inferior colliculus and the auditory cortex, both of which contain neurons selective for FM (Stiebler et al., 1997; Hage and Ehret, 2003). It is unclear whether direction selectivity can be generated in the dorsal cortex of the inferior colliculus itself. While extracellular recordings allow measuring rate and direction selectivity of neurons based on action potential firing rate, it is more difficult to assess if selectivity is generated in these cells or upstream, even though in combination with local pharmacological block of inhibition the essential role of inhibitory inputs in creating FM selectivity could be demonstrated in the bat inferior colliculus (reviewed in Fuzessery et al., 2011). This question can be more readily addressed with intracellular measurements, which allow the recording of postsynaptic responses to FM sweeps (Voytenko and Galazyuk, 2007; Gittelman et al., 2009; Gittelman and Li, 2011; Kuo and Wu, 2012). One possible approach is to compare the intracellular responses to simple tones at different frequencies and to FM sweeps in the same neuron. If the set of intracellular simple tone responses can be used to reconstruct the intracellular response to a FM sweep, it can be assumed that selectivity was generated *de novo* in this neuron by integration of synaptic inputs. In the auditory cortex, the time-shifted responses to simple tones have been compared to FM evoked responses to explore the contribution of excitation delays and spectral offsets between excitation and inhibition (Ye et al., 2010). A limitation of their approach was that the entire response to the simple tones was used, whereas an FM sweep resides only a limited time at each frequency. During FM responses, the onset responses at each frequency can be expected to be relatively important. Responses to simple tones can appear similar over a range of frequencies and sound pressure levels (SPLs), indicating the activation of a common set of inputs. Such “frequency channels” might be important for the prediction of FM evoked responses, because a FM sweep can reside for a prolonged period of time within the same channel. During this time, the same set of inputs would be activated, allowing adaptation of the response. To account for this, a prediction of FM evoked responses should put emphasis on changes and novelties in the responses to simple tones across frequencies to account for the activation of new frequency channels, while suppressing the influence of consistent responses to simple tones, reflecting the continued activation of the same frequency channel.

Here, we recorded intracellular responses to FM sweeps to study FM selectivity in the dorsal cortex of the inferior colliculus. We presented logarithmic up- and downward FM sweeps with different speeds to assess direction selectivity and rate selectivity. By comparing the responses to FM sweeps and simple tones between cells, we tested for a connection between frequency response areas (FRAs) and the responses to FM sweeps. To explore if FM selectivity can be generated in the dorsal cortex of the inferior colliculus, we reconstructed responses to FM sweeps from FRAs and we develop quantitative measures to quantify how well the reconstruction match the recorded responses.

Our data show that neurons in the dorsal cortex of the inferior colliculus can selectively respond to the direction or rate of FM sweeps. Neurons with similar responses to FM sweeps also had similar FRAs. Our reconstructions of responses to FM sweeps from FRAs suggest that FM selectivity can be generated in the dorsal cortex of the inferior colliculus. In most of the cells spectrally asymmetric inhibition appeared to be the underlying mechanism, but evidence for alternative mechanisms was also found.

## Materials and methods

A detailed description of the “Materials and Methods” used was given in Geis et al. (2011).

### Surgery

All experiments were conducted as approved by the Erasmus MC animal care ethics committee. Measurements were done on 80 C57/BL6 mice (Harlan, The Netherlands) of postnatal age between day 21 and 79. Animals were initially anesthetized with an intraperitoneal injection of ketamine–xylazine (65 and 10 mg kg^−1^). Ketamine–xylazine was supplemented as needed to reach and maintain a surgical level of anesthesia, which was assessed with the hind limb withdrawal reflex. To maintain body core temperature at 37–38°C, animals were placed on a heating pad with rectal feedback (40-90-8C; FHC, Bowdoinham, ME, USA). Eye ointment (Duratears; Alcon Nederland, Gorinchem, The Netherlands) was applied to keep the eyes moist. The skin overlying the skull was incised with a scalpel and lidocaine (Xylocaine 10%; AstraZeneca, Zoetermeer, The Netherlands) was applied to the surface before removing the bone skin. On the cleaned bone above the inferior colliculus, a titanium head plate was attached with super glue. A small hole was drilled more rostrally, above the neocortex, for the reference electrode, which was hooked between dura and bone. Both the head plate and the reference electrode were secured with dental acrylic (Simplex rapid; Associated Dental Products, Purton, UK). The bone overlying the inferior colliculus was thinned and opened via an opening in the center of the head plate. Before puncturing and deflecting the dura, bone wax was applied to the edge of the exposure. The surface of the inferior colliculus was kept moist with Ringer solution containing (in mM): NaCl 135, KCl 5.4, MgCl_2_ 1, CaCl_2_ 1.8, Hepes 5 (pH 7.2 with NaOH; Merck, Darmstadt, Germany).

### Auditory stimulation

For closed field auditory stimulation, speaker probes were inserted into the ear canals and fixed with silicon elastomer (Kwik-Cast; WPI, Berlin, Germany). Auditory stimuli were computed in MATLAB v7.0.4 (The MathWorks, Natick, MA, USA), and played back via a TDT system 3 (RP2.1 processor, PA5.1 attenuator, ED1 electrostatic speaker driver, EC1 electrostatic speaker). Intensities were calibrated for frequencies between 1 and 48.5 kHz with a condenser microphone (ACO pacific Type 7017, MA3 stereo microphone amplifier, TDT SigCal). FM sweeps had a duration of 100 ms (fast sweep) or 300 ms (slow sweep), including 2.5 ms rise/decay. Frequency was modulated logarithmically from 1 to 48.5 kHz (upward sweep) or from 48.5 to 1 kHz (downward sweep). Modulated stimuli were presented at intensities between 0 and 80 dB SPL in steps of 10 dB. We compared the responses to FM stimuli with the response to simple tones, which, apart from eight cells, had already been reported previously (Geis et al., 2011; Geis and Borst, 2013). Simple tone stimuli had durations of 100 ms, including 2.5 ms rise/decay. Frequencies between 1 and 48.5 kHz with five steps per octave were presented at intensities between 0 and 80 dB SPL in steps of 10 dB. Even at the highest intensities, we consider acoustic crosstalk between both ears unlikely to make a sizeable contribution, since recordings under similar conditions at the calyx of Held synapse, a strictly contralaterally innervated nucleus, did not show evidence for acoustic crosstalk even at high intensities (Lorteije and Borst, 2011). Stimulations were repeated 3–20 times, depending on the quality of the recording.

### Electrophysiology

*In vivo* whole-cell recordings were done under 2-photon guidance with a custom built microscope (Mai Tai laser, 800 nm; Spectra Physics Lasers, Mountain View, CA, USA) using the “shadow-patching” method (Kitamura et al., 2008), as described earlier (Geis et al., 2011). Glass pipettes (Hilgenberg, Malsfeld, Germany) with 1–2 μm tip diameter were pulled with a horizontal puller (P-97; Sutter Instrument, Novato, CA, USA) and filled with internal solution containing (in mM): potassium gluconate 126, KCl 20, Na_2_-phosphocreatine 10, Mg-ATP 4, Na_2_-GTP 0.3, EGTA 0.5, Hepes 10 (pH 7.2 with KOH; Merck, Darmstadt, Germany). The internal solution also contained 0.5% biocytin (Sigma-Aldrich, Steinheim, Germany) to retrieve cells histologically and 40 μM Alexa Fluor 594 hydrazide (Invitrogen, Carlsbad, CA, USA) to visualize cells *in vivo*. The inferior colliculus was entered with an initial pipette pressure of 30 kPa and the pressure was adjusted to 3 kPa when approaching a neuron. The brain surface was stabilized with 2% Agar (Sigma-Aldrich, Steinheim, Germany; in Ringer solution). Measurements were amplified with a MultiClamp 700A (10 kHz low pass Bessel filter), digitized with a DigiData 1322A at a sampling rate of 25 kHz, and acquired with pCLAMP 9.2 (all from Molecular Devices, Sunnyvale, CA, USA). Membrane potentials were corrected for a junction potential of −11 mV.

### Analysis

Data were analyzed with Igor pro (version 6.2.2.2; WaveMetrics, Lake Oswego, OR, USA) using NeuroMatic (version 2.00; kindly provided by Dr. J. Rothman, University College London) and custom written functions. Action potentials were detected by a threshold criterion and truncated by linearly interpolating the membrane potential 1–3 ms preceding and following the spike. The truncated responses to individual FM stimuli were correlated across repetitions (Geis et al., 2011). If the resulting autocorrelation was significantly different from zero (*p* < 0.001; *t*-test), the cell was considered responsive to this FM stimulus and we determined the properties of the response. Depending on sweep length, the number of action potentials was counted up to 150 or 350 ms after stimulus onset following a minimum delay of 7 ms after stimulus onset. Evoked rates were corrected for spontaneous firing. The spontaneous firing rate was determined in the 50 ms period before stimulus onset. Truncated membrane potential traces were averaged across all stimulus repetitions. Peak membrane potential amplitudes were detected on the smoothed, averaged membrane potential recordings in the 150 ms following the onset of a fast sweep or in the 350 ms after the onset of a slow sweep.

Selectivity indices for the FM stimuli were calculated both for action potentials and for synaptic potentials. To determine the direction selectivity index (DSI), we divided the difference in response (spikes or potential) between upward (R_up_) and downward (R_down_) sweep by the sum of the response to the two sweep directions (Britt and Starr, 1976). Positive DSI values indicate a preference for upward and negative DSI values for downward FM.

$$\text{DSI}=\frac{{\text{R}}_{\text{up}}-{\text{R}}_{\text{down}}}{{\text{R}}_{\text{up}}+{\text{R}}_{\text{down}}}$$

The rate selectivity index (RSI) was calculated by subtracting the ratio between the mean response (R_mean_) and the maximal response (R_max_) from 1, and multiply by 2 to have RSI values ranging from 0 (no rate preference) to 1 (high rate preference) (Brown and Harrison, 2009).

$$\text{RSI}=2\times \left(1-\left(\frac{{\text{R}}_{\text{mean}}}{{\text{R}}_{\text{max}}}\right)\right)$$

To compare the responsiveness to modulated and simple stimuli between neurons, we concatenated the measured membrane potentials of the whole FRA of a cell into one vector and the membrane potential in response to all FM sweeps into another vector. Each FRA vector had a total duration of 261 s, consisting of a total of 522 different 100 ms stimuli (29 different frequencies, at nine different intensities presented to both the contra- and ipsilateral ear), at an interstimulus interval of 400 ms. The FM vectors had a total duration of 36 s, containing contiguous 500 ms recordings segments of stimulations with four different FM sweeps (up, down, slow, and fast), at nine different intensities, presented to both the contra- and the ipsilateral ear. Stimulus duration was either 100 or 300 ms, depending on sweep speed. FRA and FM vectors were created for each repetition of the whole stimulus set and cross-correlated to all the corresponding vectors in another cell, excluding correlations of identical repetition number. The resulting cross-correlation values were averaged over repetitions, summarizing the similarity of responses to simple tones or FM sweeps between two neurons in one correlation value.

### Reconstruction

We employed three different models to reconstruct responses to FM sweeps from responses to the simple tones used in the FRA. The simplest model, called “Delay,” assumes only a time delay from the onset of the FM sweep until it reaches a specific frequency. Subthreshold potentials in response to simple tones were time shifted and averaged. Since we presented logarithmic FM sweeps and simple tones with logarithmic frequency spacing, for the reconstruction a linear increase in delay between simple tone responses was assumed. Delays for time shifting were therefore calculated by dividing the FM sweep duration by the number of simple tones. By linearly integrating the delay shifted responses to simple tones over the range from 1 to 48.5 kHz, this reconstruction does not take any temporal or spectral non-linearities into account, which might contribute to the generation of FM selective responses.

Our second model used the same time delay, but, in addition, emphasizes the onset response generated by simple tones. We only recorded responses to simple tones with a duration of 100 ms, while the FM sweeps we used passed through the entire frequency range between 1 and 48.5 kHz in 100 or 300 ms total. To reduce the impact of the late response during the response to a 100 ms simple tone and to increase the impact of onset responses, we convoluted subthreshold potential changes with an impulse function of the form e^(−(ln(t/t_onset_)/width)^2^)^, where t was time, t_onset_ the average postsynaptic potential onset time for the given SPL and width was set to values of 0.12 for short and 0.32 for long sweeps. In this “Onset” model, after convolution the responses were time shifted, summed and scaled according to the overlap between the impulse function after time shift.

With our third model, called “Channel,” we tried to highlight the influence of different frequency channels on the response to FM sweeps. FRAs contain areas with uniform responses to simple tones of different frequencies, suggesting that the cell receives the same set of inputs for all frequencies within that area. A FM sweep passing through such a frequency channel would activate the same inputs for a prolonged period of time, potentially leading to a reduced response due to prolonged activation. Assuming such an adaptation of the response if a FM sweep moves over frequencies within one frequency channel, and increased responsiveness if a FM sweep enters a new frequency channel, in our reconstruction we only used responses to simple tones if they were larger or if they were sufficiently different from the preceding simple tone responses in the FM reconstruction. Responses were considered larger if a line fit that was constrained to go through the origin in the graph in which the response that came later in the reconstruction is plotted against the earlier resulted in a slope bigger than one. Responses were considered different if the Pearson correlation between the earlier and the later response was less than 0.92. If responses were larger or sufficiently different, the difference between the two time-shifted responses was added to the reconstruction. If this was not the case, the reconstruction was scaled down using a vector. To get this vector, the two tone responses were subtracted after appropriate time shifting. The difference was divided by the maximum size of the absolute response to the earlier tone, clipped to a maximum value of one, after which it was subtracted from 1 and multiplied with the running sum. This strategy ensured that downscaling was proportional to the difference in the responses to the two simple tones.

## Results

We made whole-cell recordings from a total of 123 neurons in the dorsal cortex of the inferior colliculus. Their average resting membrane potential was −66 ± 1 mV. The median minimum threshold for postsynaptic potentials was 40 dB SPL. To determine their FM selectivity, we measured their response to logarithmic FM sweeps. A total of 121 neurons showed a consistent synaptic response to at least one FM stimulus. In 31 of these cells only contralateral FM stimulation evoked spikes, in 8 cells only ipsilateral, and in the remaining 26 cells both contra- and ipsilateral FM stimulation evoked spikes.

### Direction selective neurons in the dorsal cortex of the inferior colliculus

**Figure 1:**
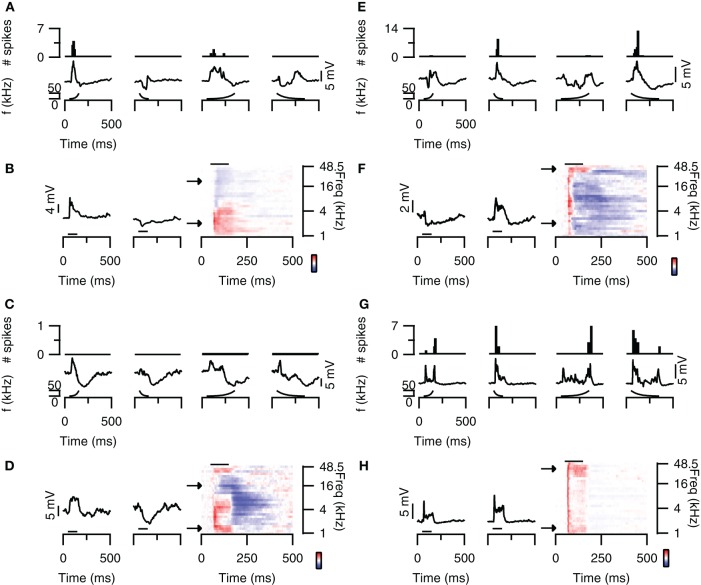
**FM direction selectivity in the dorsal cortex of the inferior colliculus. (A)** Responses of an upward direction selective neuron. Poststimulus time histograms are shown in the top panel, underlying membrane potential changes in the bottom panel. Resting membrane potential was −64 mV. **(B)** Responses to 80 dB SPL tones of the neurons in **(A)** on the right with two example traces corresponding to the responses to simple tones at the frequencies indicated by the arrows. Color bar indicates ±13 mV. Horizontal bars mark the stimulus. **(C)** Responses of an EPSP-based upward selective neuron that did not fire action potentials. Layout as in **(A)**. Resting membrane potential was −69 mV. **(D)** Responses to 80 dB SPL tones of the neurons in **(C)** on the right with two example traces corresponding to the responses to simple tones at the frequencies indicated by the arrows. Color bar indicates ±12 mV. **(E)** Responses of a spike-based downward direction selective neuron. Layout matches **(A)**. Resting membrane potential was −64 mV. **(F)** Responses to 80 dB SPL tones of the neurons in **(E)** on the right with two example traces corresponding to the responses to simple tones at the frequencies indicated by the arrows. Color bar indicates ±5 mV. **(G)** Responses of a direction unselective neuron. Layout as in **(A)**. Resting membrane potential was −61 mV. **(H)** Responses to 80 dB SPL tones of the neurons in **(G)** on the right with two example traces corresponding to the responses to simple tones at the frequencies indicated by the arrows. Color bar indicates ±13 mV. Stimuli were presented to the contralateral ear at 80 dB SPL.

About 20% of neurons in the dorsal cortex of the inferior colliculus showed selectivity to the direction of FM sweeps. Three examples are shown in Figures 1A–F. In response to 80 dB FM stimuli presented to the contralateral ear, the cell shown in Figure 1A fired action potentials to upward fast or slow modulated sweeps, but not to downward (Figure 1A; top row). This firing behavior resulted in a DSI for spikes of +1 for both fast and slow sweeps. Upward FM sweep also evoked larger EPSPs while downward FM sweeps evoked a larger hyperpolarization (Figure 1A; bottom row). The difference in EPSP amplitude is reflected in the positive EPSP based DSI values of 0.56 for fast sweeps and 0.18 for slow sweeps.

A relatively frequently occurring mechanism underlying upward direction selective responses was the combination of low frequency excitation and high frequency inhibition. The responses to 80 dB SPL tones of the neuron displayed in Figure 1A show clear excitatory and inhibitory frequency areas (Figure 1B; right). At low frequencies (bottom arrow, left trace), the neuron responded with an EPSP to stimulation with a simple 100 ms tone (horizontal bar) presented to the contralateral ear at 80 dB SPL. In contrast, at high frequencies (top arrow, right trace) the stimulation evoked an IPSP. Another example of an upward direction selective neuron is shown in Figures 1C and D. This cell responded with IPSPs and subthreshold EPSPs to FM stimuli. EPSPs were largest for fast upward modulated sweep and very small for fast downward modulated sweeps. This difference resulted in a DSI of 0.62, classifying this cell as upward selective. Similar to the neuron showed in Figures 1A,B, the responses to 80 dB SPL tones of this neuron also showed low frequency excitation and high frequency inhibition (Figure 1D).

Whereas the combination of high frequency inhibition and low frequency excitation could underlie upward FM selective responses, the reverse could underlie downward FM selective responses. The neuron in Figure 1E fired more action potentials and showed larger EPSPs in response to downward FM sweeps. Based on a spike based DSI of −0.75 for fast and −0.8 for slow sweeps, as well as an EPSP based DSI of −0.32 for fast and −0.58 for slow sweeps, this cell was classified as downward FM selective. The responses to 80 dB SPL tones of this neuron showed inhibitory and excitatory frequency areas, with inhibition dominating at low frequencies and excitation more prominent at high frequencies (Figure 1F).

The majority of the neurons in the dorsal cortex showed little selectivity for the direction of FM sweeps. An example of a cell that was not selective for the direction of FM sweeps is shown in Figure 1G. This neuron had a DSI for spikes of −0.26 for fast FM sweeps and of −0.18 for slow sweeps, whereas DSIs for EPSPs were −0.13 and −0.14 for fast and slow sweeps, respectively. The responses to 80 dB SPL tones showed an EPSP, which was longer lasting at both the lowest and the highest frequencies (Figure 1H), providing an explanation for the two-peaked synaptic response during FM sweeps. The response to a fast downward modulated sweep did not show a clear second peak, probably owing to the larger delayed EPSP evoked at high frequencies.

**Figure 2:**
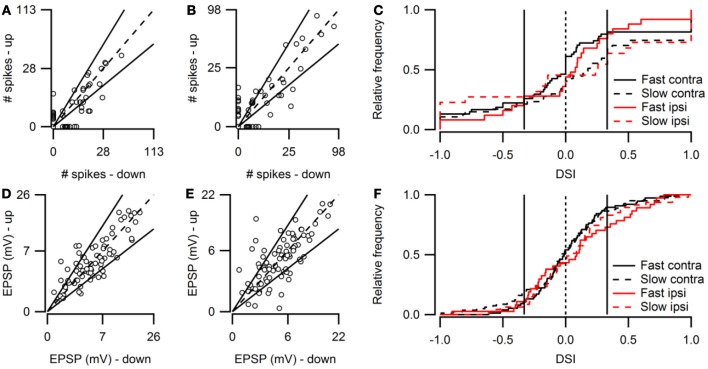
**Overview of FM direction selectivity. (A)** Number of action potentials evoked by fast upward sweeps plotted against the number of spikes in response to fast downward sweeps presented to the contralateral ear (*n* = 84; 36 cells at origin). Solid lines next to identity line indicate DSI of ±0.33. **(B)** Action potentials fired in response to slow up- and downward sweeps presented to the contralateral ear (*n* = 88; 41 cells at origin). **(C)** Cumulative distribution of spike-based DSIs in response to fast or slow FM sweeps presented to the contra- or ipsilateral ear. Vertical solid lines indicate a DSI of ±0.33. **(D)** EPSP amplitudes in response to fast upward sweeps plotted against amplitudes in response to fast downward sweeps presented to the contralateral ear (*n* = 76). Solid lines indicate DSI of ±0.33. **(E)** EPSP amplitudes evoked by slow up- and downward sweeps presented to the contralateral ear (*n* = 81). **(F)** Cumulative distribution of EPSP amplitude based DSIs in response to fast or slow FM sweeps presented to the contra- or ipsilateral ear. Vertical solid lines indicate a DSI of ±0.33.

Figure 2 shows population data for direction selectivity. In most neurons the number of action potentials fired in response to contralateral stimulation with fast up- or downward FM sweeps was similar (Figure 2A). The two solid lines in Figure 2A indicate a DSI of +0.33 or −0.33, which we used as the cutoff for selectivity. Twenty percent of neurons showed direction selectivity for fast sweeps during contralateral stimulation. The majority of these cells (67%) lined up along the axes, indicating a DSI of +1 or −1. The number of up- and downward selective neurons was similar (Figure 2C; Table 1). A slightly larger percentage of cells (24%) showed direction selectivity in response to slow FM sweeps presented to the contralateral ear (Figure 2B). Of these, slightly more than half (55%) responded exclusively to the preferred direction. More than half (55%) of the neurons that showed direction selectivity to slow sweeps preferred upward sweeps (Table 1).

**Table 1:** **Direction and rate selectivity in the dorsal cortex of the inferior colliculus**.

		**Direction selectivity**		**Rate selectivity**	
		**Fast sweep**	**Slow sweep**	**Up sweep**	**Down sweep**
Spikes	Contralateral	20%	24%	14%	17%
		10up/14down	16up/13down	5fast/12slow	10fast/11slow
	Ipsilateral	13%	10%	11%	12%
		7up/9down	8up/4down	6fast/7slow	7fast/8slow
EPSPs	Contralateral	13%	21%	6%	7%
		8up/8down	11up/15down	3fast/4slow	4fast/4slow
	Ipsilateral	11%	10%	7%	7%
		10up/4down	8up/4down	2fast/7slow	3fast/6slow

*Percentage of direction and rate selective neurons for different sets of FM sweeps, as determined by the number of action potentials or the EPSP peak amplitude. FM stimuli were presented to the contra- or ipsilateral ear at 80 dB SPL*.

Fewer cells showed direction selectivity in response to ipsilateral stimulation (Table 1), but the overall distribution of DSIs was similar for ipsi- and contralateral stimulation (Figure 2C). In response to fast FM sweeps presented to the ipsilateral ear, 13% of cells were direction selective. About one third of these fired action potentials only to the preferred direction. No preferred direction was apparent (Table 1). Slow FM sweeps presented to the ipsilateral ear revealed 10% direction selective neurons, and 67% of these fired spikes only to sweeps in the preferred direction. Again, there was a somewhat higher percentage of upward than downward selective neurons (Table 1), suggesting a slight preference for upward modulation in the case of slow sweeps, regardless of the side of stimulation.

Based on EPSP amplitude, 13% of neurons in the dorsal cortex of the inferior colliculus showed direction selectivity to stimulation with fast and 21% to stimulation with slow FM sweeps. The peak amplitudes of the EPSPs in response to fast up- and downward FM sweeps presented to the contralateral ear are shown in Figure 2D. Most data points cluster around the unity line, indicating a low percentage of direction selective neurons. The number of neurons responding with larger EPSPs to fast up- or downward sweeps was similar, indicating no clear FM sweep direction preference in response to fast sweeps (Table 1). Slow sweeps evoked selective responses more often, with 21% of cells showing EPSP based direction selectivity (Figure 2E). There was a slight preference for downward sweeps (Table 1).

The distributions of DSIs resulting from stimulation with slow or fast FM sweeps presented to the ipsilateral ear were similar as for the contralateral ear (Figure 2F). A shift of the distribution to higher direction selectivity values, suggesting a preference for upward sweeps, was observed only for fast FM sweeps presented to the ipsilateral ear. Stimulation of the ipsilateral ear with fast FM sweeps revealed that 11% of cells were direction selective (Table 1). Of these, 71% preferred upward modulated sweeps. Similar results were obtained for slow FM sweeps presented to the ipsilateral ear; 10% of cells were direction selective, with a preference for upward sweeps (Table 1).

**Figure 3:**
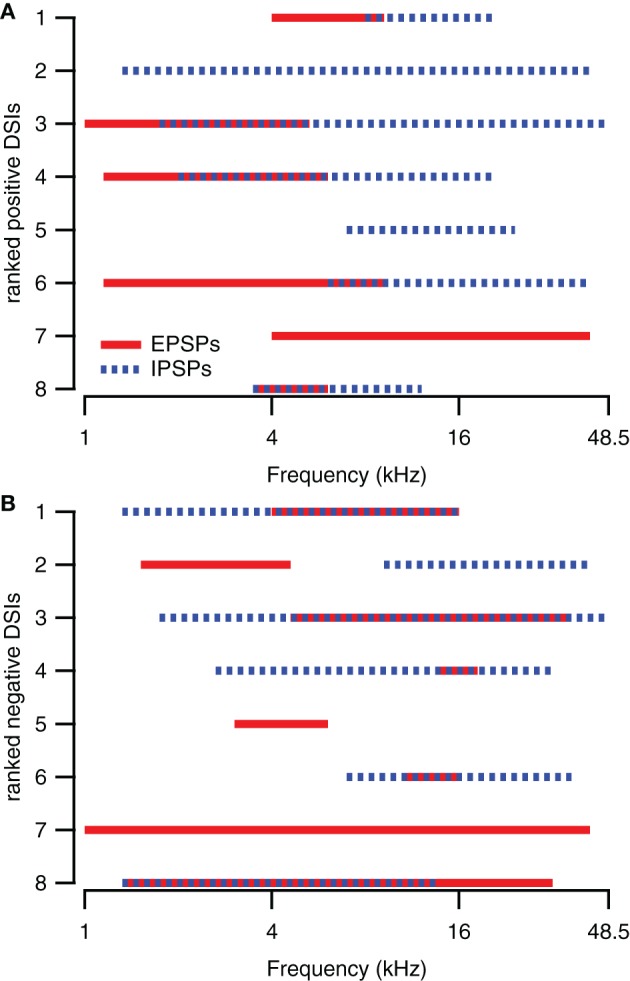
**Spectral integration of excitatory and inhibitory areas underlies direction selectivity in about half of the direction selective neurons. (A)** Excitatory and inhibitory response areas of upward direction selective neurons (DSI >0.33) determined at 80 dB SPL. **(B)** Excitatory and inhibitory response areas of downward direction selective neurons (DSI <−0.33) determined at 80 dB SPL. The smallest rank was given to the neuron with the largest absolute DSI value.

The presence of excitatory and inhibitory areas in response to 80 DB SPL tones of different frequencies can explain direction selective responses in about half of the direction selective neurons. Of the eight upward direction selective neurons, five had responses to 80 dB SPL tones with excitatory areas at lower frequencies than their inhibitory areas (Figure 3A), similar to the cell shown in Figures 1A,B. Of the eight downward direction selective neurons, four had responses to 80 dB SPL tones with excitatory areas at higher frequencies and inhibitory areas extending to lower frequencies (Figure 3B), similar to the neuron shown in Figures 1E,F. Of the remaining seven cells, three had responses to 80 dB SPL tones with only excitatory areas (Figure 1G), two neurons showed only inhibitory areas and two cells showed a mix of excitatory and inhibitory areas that did not match their direction selectivity. In these seven cells, the direction selective response either was inherited from upstream nuclei, or it followed from a spectrotemporal interaction of excitatory and/or inhibitory conductances other than the mechanism that generated direction selectivity in the cells shown in Figures 1A–F.

### Rate selective neurons in the dorsal cortex of the inferior colliculus

**Figure 4:**
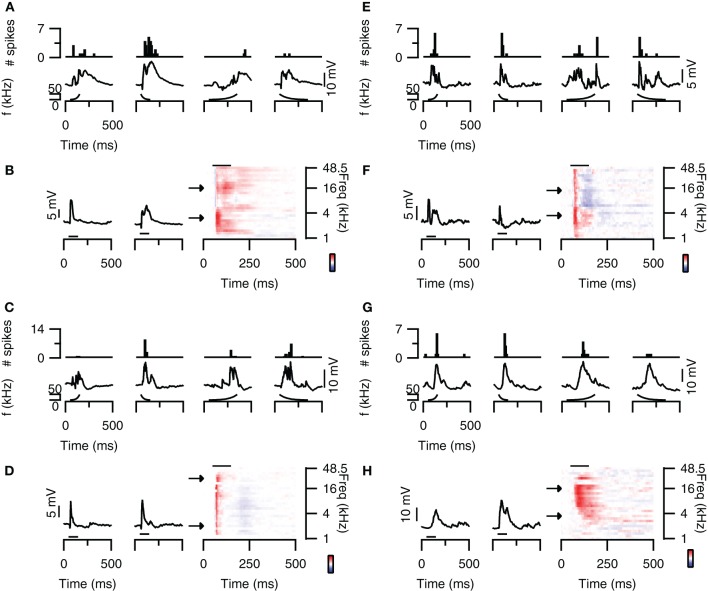
**FM rate selectivity. (A)** Neuron with fast rate selectivity for downward sweeps. Poststimulus time histograms are shown in the top panel, underlying membrane potential changes in the bottom panel. Resting membrane potential was −58 mV. **(B)** Responses to 80 dB SPL tones of the neurons in **(A)** on the right with two example traces corresponding to the responses to simple tones at the frequencies indicated by the arrows. Color bar indicates ±15 mV. **(C)** Neuron with spike-based slow rate selectivity for upward sweeps. Layout matches **(A)**. Resting membrane potential was −68 mV. **(D)** Responses to 80 dB SPL tones of the neurons in **(C)** on the right with two example traces corresponding to the responses to simple tones at the frequencies indicated by the arrows. Color bar indicates ±15 mV. **(E)** Responses of a rate unselective neuron with EPSPs and IPSPs in the responses to 80 dB SPL tones. Layout as in **(A)**. Resting membrane potential was −65 mV. **(F)** Responses to 80 dB SPL tones of the neurons in **(E)** on the right with two example traces corresponding to the responses to simple tones at the frequencies indicated by the arrows. Color bar indicates ±9 mV. **(G)** Responses of a rate unselective neuron with a mainly excitatory responses to 80 dB SPL tones. Layout as in **(A)**. Resting membrane potential was −65 mV. **(H)** Responses to 80 dB SPL tones of the neurons in **(G)** on the right with two example traces corresponding to the responses to simple tones at the frequencies indicated by the arrows. Color bar indicates ±22 mV. Stimuli were presented to the contralateral ear at 80 dB SPL.

Some neurons in the dorsal cortex of the inferior colliculus showed rate selectivity for FM sweeps. Figure 4A (top) shows an example of a cell that mainly fired action potentials in response to fast sweeps. The spike based rate selectivity index was 1 for upward sweeps and 0.93 for downward sweeps. EPSP-based RSIs were much smaller in this cell, but followed the same trend with values of 0.07 for upward sweeps and 0.39 for downward sweeps (Figure 4A; bottom). The responses to 80 dB SPL tones of this cell were dominated by excitation, while weak onset inhibition was present at higher frequencies (Figure 4B). This inhibition might explain the preference for downward sweeps in this cell, as downward sweeps would first pass through an inhibitory area before entering an excitatory area. This observation, however, does not offer a basis for the observed rate selectivity, as the high frequency onset inhibition appears transient regardless of the downward sweep speed (Figure 4A; bottom panel). Instead, the time course of the EPSPs might offer an explanation for the rate selectivity, because at low frequencies EPSPs were more transient with shorter delays, whereas at higher frequencies EPSPs had longer delays and durations. These EPSPs are therefore expected to coincide during fast downward sweeps.

An example of an upward rate selective neuron is shown in Figure 4C (top). In this neuron the spike-based RSI was much larger for upward sweeps (0.71) than for downward sweeps (0.06). EPSP amplitudes also differed most between fast and slow upward sweeps, which led to RSIs of 0.40 for upward sweeps and 0 for downward sweeps (Figure 4C; bottom). The responses to 80 dB SPL tones of this neuron appeared fairly uniform across frequencies (Figure 4D). EPSPs were preceded by onset IPSPs and often followed by a late, slight hyperpolarization. In this rate selective neuron, the response to tones thus did not offer a good explanation for the rate selectivity.

Most neurons in the dorsal cortex of the inferior colliculus did not show FM rate selectivity. An example is shown in Figure 4E. Action potentials were elicited at fast and slow sweep rate, resulting in a RSI of 0 for upward and of 0.09 for downward sweeps (Figure 4E; top). EPSP amplitudes were also similar between sweep rates, resulting in EPSP-based RSI values of 0.05 for upward and 0.22 for downward sweeps (Figure 4E; bottom). The responses to 80 dB SPL tones showed onset excitation across most frequencies, which was followed by delayed excitation at low frequencies and delayed inhibition at higher frequencies (Figure 4F). Whereas the interaction between onset and delayed responses would represent a good substrate for rate selectivity in this neuron, we did not observe rate selective responses at the rates tested. Another example of a neuron that did not show rate selectivity is displayed in Figure 4G. This cell fired action potentials in response to fast and slow upward as well as downward sweeps (Figure 4G; top). While the number of spike differed with sweep rate, the RSIs reached only 0.44 for upward sweeps and 0.5 for downward sweeps. EPSP amplitudes were even more similar, resulting in EPSP-based RSIs of 0.18 for upward and 0.04 for downward sweeps (Figure 4G; bottom). The responses to 80 dB SPL tones mainly showed excitatory inputs, which had longer delays at low frequencies than at high frequencies (Figure 4H). This frequency-dependent difference in delays could offer a mechanism for rate selectivity, except that we did not observe a rate selective response in this neuron at the rates tested.

**Figure 5:**
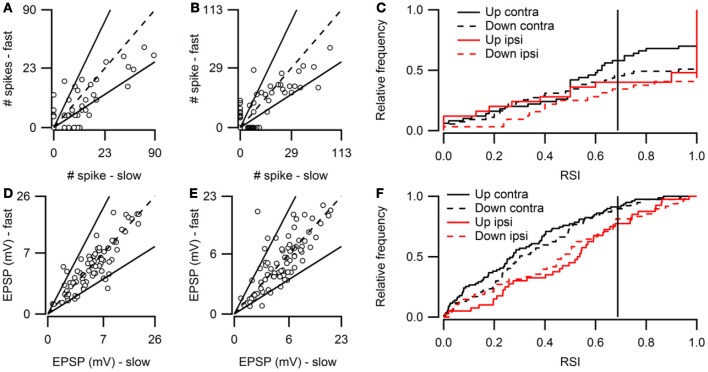
**Overview of FM rate selectivity. (A)** Number of action potentials evoked by contralateral fast upward sweeps plotted against the number of spikes in response to contralateral slow upward sweeps (*n* = 86; 49 cells at origin). Solid lines next to identity line indicate RSI of 0.6875. **(B)** Action potentials fired in response to fast and slow downward sweeps presented to the contralateral ear (*n* = 85; 43 cells at origin). **(C)** Cumulative histogram of spike-based RSIs in response to up- or downward FM sweeps presented to the contralateral or ipsilateral ear. Vertical solid line indicates a RSI of 0.6875. **(D)** EPSP amplitudes in response to contralateral fast upward sweeps plotted against amplitudes in response to contralateral slow upward sweeps (*n* = 77). Solid lines indicate RSI of 0.6875. **(E)** EPSP amplitudes evoked by fast and slow downward sweeps presented to the contralateral ear (*n* = 79). **(F)** Cumulative histogram of EPSP amplitude based RSIs in response to up- or downward FM sweeps presented to the contralateral or ipsilateral ear. Vertical solid line indicates a RSI of 0.6875.

Figure 5 provides population data for FM rate selectivity, illustrating that the majority of neurons in the dorsal cortex did not show rate selectivity for the rates tested. Based on the number of action potentials, 14% of neurons in the dorsal cortex of the inferior colliculus were selective for the rate of upward FM sweeps presented to the contralateral ear. In Figure 5A these cells are positioned outside the solid lines that indicate a RSI value of 0.6875. Fifty-nine percent of upward rate selective neurons fired action potentials only to the preferred sweep rate, resulting in an RSI of +1 and a position along one of the axes. Twenty-nine percent of upward rate selective neurons preferred fast sweep rates presented to the contralateral ear (Table 1), leading to a position close to the abscissa in Figure 5A.

Based on action potential firing, 17% of cells were selective for the rate of a downward FM sweep presented to the contralateral ear (Figure 5B). Of these, 81% responded only to one sweep rate (Figure 5C). About half of downward sweep rate selective neurons preferred fast sweep rates. When stimuli were presented to the ipsilateral ear, 11% of cells showed rate selectivity with upward sweeps and 12% with downward sweeps (Table 1). Most of these rate selective cells responded to only a single FM rate (upward: 85%, downward: 87%).

Based on EPSP amplitude, only few neurons were rate selective for FM sweeps. Six percent of neurons were selective for the rate of upward sweeps presented to the contralateral ear (Figure 5D). Little rate selectivity (7%) was also observed for EPSP amplitudes when downward sweeps were presented to the contralateral ear (Figure 5E). RSI distributions were slightly shifted toward higher values for ipsilateral stimulation compared to contralateral stimulation (Figure 5F). Ipsilaterally presented sweeps evoked selective responses in 7% of neurons when modulated either up- or downward (Table 1).

### Correlation between supra- and subthreshold selectivity indices

**Figure 6:**
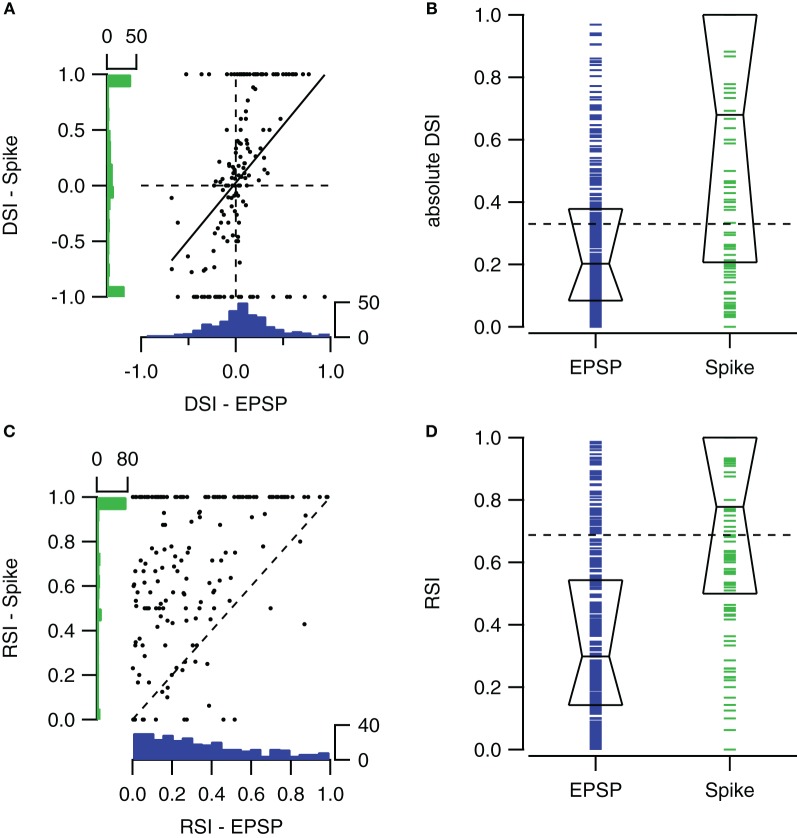
**Comparison of sub- and suprathreshold selectivity indices. (A)** Scatter plot showing the relation between the fast sweep DSI for the number of action potentials and for EPSPs within the same cells for all sound intensities ranging from 0 to 80 dB SPL (*n* = 153 from 57 cells). Solid line shows the regression line (*r* = 0.42). At left and bottom the histograms of spike- and EPSP-DSI are shown. **(B)** Fast sweep DSIs for spikes (*n* = 160) are generally larger than for EPSP amplitudes (*n* = 302). Boxes indicate the median with first and third quartile. **(C)** Upward sweep RSIs for number of action potentials and for EPSPs (*r* = 0.38; *n* = 172 from 55 cells). **(D)** Upward sweep RSIs for spikes (*n* = 181) are generally larger than for EPSP amplitudes (*n* = 330). Boxes indicate the median with first and third quartile. Selectivity indices were determined using contralateral stimuli ranging from 0 to 80 dB SPL. EPSP-based DSIs and RSIs were only included when autocorrelation of the two underlying postsynaptic potentials were significant. Spike-based selectivity indices were only included if the cell fired in response to at least one of the two FM stimuli.

Spike-based and EPSP-based DSIs were not very well correlated (*r* = 0.42). Figure 6A compares spike- and EPSP-based DSIs for contralateral stimulation ranging between 0 and 80 dB SPL. The distribution of EPSP-based DSIs was centered around zero, showing only few values close to +1 or −1. The distribution of spike-based DSIs was also centered around zero, but extreme values of −1 and +1 were more common.

The spike-based DSI was generally larger than the EPSP-based DSI (median DSI: 0.20 vs. 0.68, Mann–Whitney U = 36087, *n*_1_ = 160, *n*_2_ = 302, *P* < 0.001 two-tailed; Figure 6B).

Spike-based and EPSP-based RSIs showed little correlation (Figure 6C). Membrane potential based RSIs were generally low. The distribution was skewed to the right. In contrast, action potential based RSIs were distributed more evenly, except for a large peak at the maximum value of +1. This peak is responsible for the large difference between the medians of the two RSI indices (0.30 vs. 0.78, Mann–Whitney U = 52786, *n*_1_ = 218, *n*_2_ = 330, *P* < 0.001 two-tailed; Figure 6D).

### FM responses can often be predicted by tone responses

**Figure 7:**
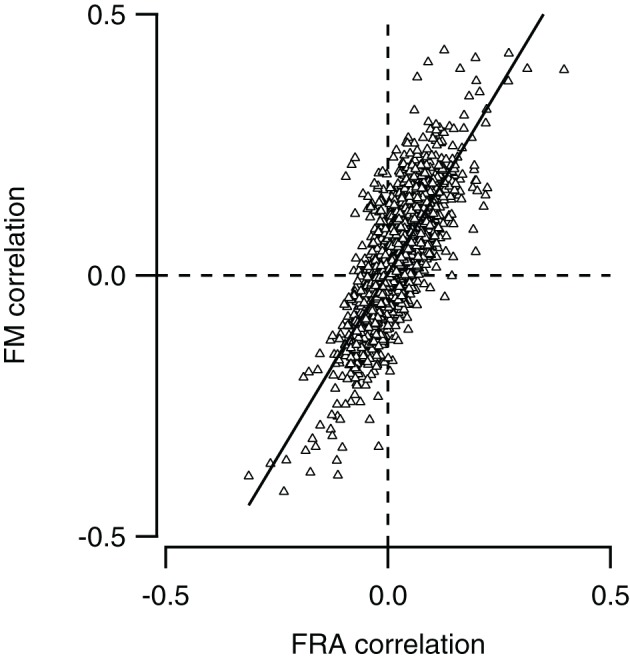
**Neurons with similar frequency response areas also respond similarly to FM sweeps.** The Pearson correlation between the FM responses of any pair of neurons (FM correlation) is plotted against the Pearson correlation of the frequency response area as of the same pair (FRA correlation). Line shows a line fit though the data (*r* = 0.77).

We compared for each neuron pair in our dataset how similar responses to simple tones were, and compared this with the similarity of responses to FM sweeps. To calculate how similar tone responses were, we lumped together the membrane potential measurements during stimulation with all 29 simple tones presented at nine different SPLs to the contra- and ipsilateral ear, constituting the FRA. Correlation was then calculated as described in the “Materials and Methods.” We also combined recordings during stimulation with the four types of FM sweeps presented at nine SPLs to the contra- and ipsilateral ear. Comparing the correlations in response to stimulation with simple tones and FM sweeps allowed us to explore if neurons with similar FRAs also showed similar responses to FM sweeps. As shown in Figure 7, the two correlations correlated well (*r* = 0.77), suggesting that the responses to FM sweeps can be predicted from the FRA of a neuron.

### Use of three methods to predict FM responses from simple tone responses

Two pieces of evidence suggest that FM selectivity can be generated within the dorsal cortex. Firstly, the spectral separation of inhibition and excitation in a subset of cells provides a well-known mechanism for the creation of direction selectivity (reviewed in Fuzessery et al., 2011; Pollak et al., 2011). Secondly, the observation that neurons with similar responses to tones also have similar responses to FM sweeps suggests that in many more neurons, the responses to FM sweeps are determined by the integration of synaptic inputs, rather than inherited from upstream structures. However, the positive correlation observed in Figure 7 does not contain proof that responses are locally generated, since two neurons that inherit the same type of FM response may also inherit the same type of tone response. In addition, especially for the rate selective neurons, the underlying mechanism for the difference in the responses to fast and slow sweeps was generally not obvious in our dataset. To more directly investigate how well responses to FM can be predicted from the FRAs, we therefore tried to directly reconstruct FM responses from the responses to simple tones.

**Figure 8:**
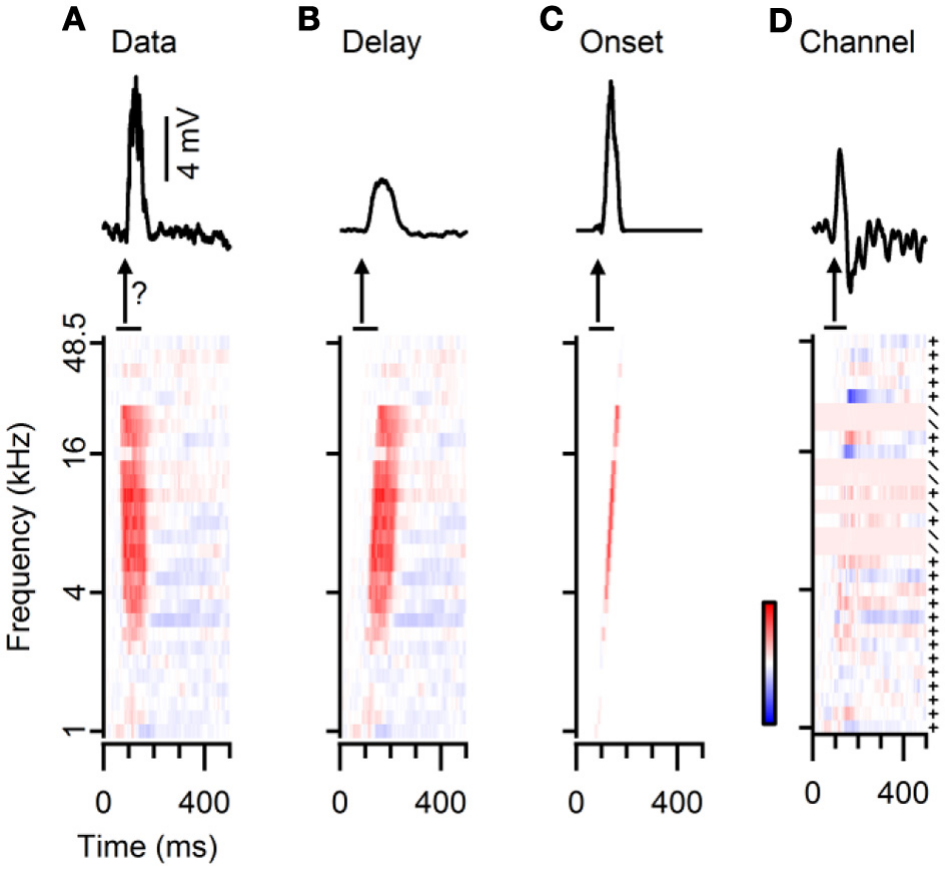
**Three different methods for reconstructing responses to FM sweeps from the responses to simple tones. (A)** Average subthreshold potential responses to a fast upward FM sweep (top) and to simple tones (bottom). The question mark indicates that the goal of our reconstructions is to reproduce FM responses from tone responses. **(B)** “Delay” method: responses to simple tones were time shifted (bottom), followed by averaging to obtain the reconstruction (top). **(C)** “Onset” method: responses to simple tones were convoluted with an impulse function and time shifted (bottom) to create the reconstruction (top). **(D)** “Channel” method: differences in responses to simple tones of increasing frequencies were either added (+) or used as a scaling factor (/) to reconstruct the FM model (top). Color bar indicates ±10 mV.

We used three different reconstruction methods, named “Delay,” “Onset,” and “Channel,” as described in the “Materials and Methods” section. Figure 8A shows the responses to 80 dB SPL tones of a neuron together with the response to a fast upward FM sweep at the same sound intensity. In addition, the graph illustrates how the responses to 80 dB SPL tones were modified to calculate the three different reconstructions. In the “Delay” method merely an incremental delay was applied (Figure 8B). In the “Onset” method the same delay was applied, but only the onset of each component was used (Figure 8C). The “Channel” method used either addition or scaling, depending on the relative changes in the response to simple tones of neighboring frequencies (Figure 8D). All three reconstructions reproduced the excitatory nature of the FM evoked response in this example and captured some aspects of the EPSP timing. The reconstructions also exemplify some of the limitations of the three reconstruction methods. A relatively minor problem was the scaling of the reconstructions. In this example, the amplitudes of the Delay and of the Channel reconstructions were smaller than of the measured response (Figures 8B,D). This only hampers a comparison of EPSP amplitudes between different types of reconstructions, whereas a comparison within one reconstruction method and the calculation of DSI and RSI was not affected. A more specific issue concerning the Delay reconstructions can be seen in Figure 8B, as the reconstructed EPSP appears slightly delayed and broader than the recorded response to the FM stimulus. A slight delay was also introduced in the Onset reconstruction, but, by focusing on the onset responses to each simple tone, the reconstructed EPSP was not broadened and its amplitude matched that of the FM evoked response (Figure 8C). A drawback of the Channel reconstruction method was the relatively strong baseline fluctuations due to the scaling of the trace compared to the averaging done in the other reconstructions (Figure 8D). In addition, in this example an IPSP was artificially introduced that resulted from the difference between a simple tone stimulus that elicited an EPSP and one that did not elicit an obvious response. Compared to the Delay and the Onset reconstruction, the Channel reconstruction described the onset time of the response best. Owing to the averaging of time shifted responses, the amplitudes of our reconstructions often deviated from the amplitudes of FM evoked responses.

### Reconstruction of direction selective fm responses from simple tone responses

**Figure 9:**
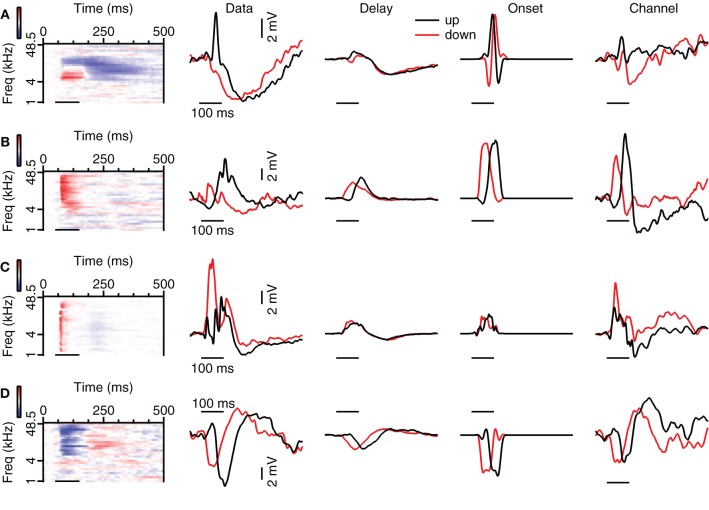
**Examples of reconstructions of up- and downward FM sweeps. (A)** Neuron with strong correlation values for the Channel reconstructions, but weaker correlations for the Delay and Onset reconstructions. From left to right: responses to 80 dB SPL tones, averaged subthreshold response to fast FM sweep at 80 dB SPL (“Data”), Delay, Onset, and Channel reconstructions. Correlation values during stimulation: 0.11 (up Delay), −0.11 (down Delay), −0.34 (up Onset), −0.12 (down Onset), 0.97 (up Channel), 0.61 (down Channel). Selectivity error: 1.78 mV (Delay), 3.28 mV (Onset), 1.34 mV (Channel). Resting membrane potential was −71 mV. **(B)** Neuron with strong correlations for upward sweeps. Correlation values during stimulation: 0.89 (up Delay), 0.10 (down Delay), 0.96 (up Onset), 0.06 (down Onset), 0.97 (up Channel) and 0.67 (down Channel). Selectivity error: 2.10 mV (Delay), 2.94 mV (Onset), 1.66 mV (Channel). Resting membrane potential was −74 mV. **(C)** Neuron with variable correlations for the types of reconstructions. Correlation values during stimulation: 0.46 (up Delay), −0.0.33 (down Delay), 0.47 (up Onset), 0.56 (down Onset), −0.19 (up Channel), 0.79 (down Channel). Selectivity error: 2.94 mV (Delay), 4.78 mV (Onset), 0.13 mV (Channel). Resting membrane potential was −68 mV. **(D)** Neuron showing good correlations for most reconstruction methods. Correlation values during stimulation: 0.92 (up Delay), 0.34 (down Delay), 0.83 (up Onset), 0.70 (down Onset), 0.85 (up Channel) and 0.91 (down Channel). Selectivity error: 1.37 mV (Delay), 1.61 mV (Onset), 1.95 mV (Channel). Resting membrane potential was −58 mV.

We then compared the predictions from the three models based on the responses to 80 dB SPL tones with the recorded FM responses. In some cells with spectrally separated excitatory and inhibitory tone response areas, reconstructions could reproduce FM responses well. An example is the neuron shown in Figure 9A, which responded with an EPSP to upward FM sweeps that was absent in response to downward sweeps. The responses to 80 dB SPL tones showed a low frequency excitatory area neighboring a high frequency inhibitory area. This configuration would allow the cell to respond with an EPSP followed by an IPSP to upward sweeps while a downward sweep would let the EPSP coincide with the IPSP. Our Delay and Channel reconstruction showed that the EPSP coincided with the IPSP during the upward sweep, while there was only a minor deflection in the response to the downward sweep. The overestimation of the EPSP in our simple reconstructions of responses to downward sweeps suggests that the impact of inhibition was underestimated. Despite these imperfections, FM direction selective responses could be reproduced with a method that did not require any free parameters, suggesting that the direction selectivity of this cell was generated locally in the dorsal cortex of the inferior colliculus based on the asymmetry of excitation and inhibition in its responses to 80 dB SPL tones. In a total of 7 of 9 fast sweep direction selective neurons that displayed asymmetric excitation and inhibition in response to 80 dB SPL tones direction selectivity was reproduced in at least one of the reconstructions. The same was true for 5 of 9 slow sweep direction selective cells that showed asymmetric excitation and inhibition.

The reconstructions also successfully reproduced FM responses in cells for which EPSP onset depended on tone frequency. The cell shown in Figure 9B responded with a larger EPSP to upward than to downward sweeps. This response behavior was also reflected in our reconstructions, indicating that the responses to 80 dB SPL tones contain the essential information required for direction selectivity generation. As the responses to 80 dB SPL tones in this cell were dominated by excitation, the interaction of excitatory inputs with different delays is the most parsimonious explanation for the observed direction selectivity. EPSPs delays appeared shortest at high frequencies and longer at lower frequencies. In this cell an upward sweep would first activate a long-latency EPSP, allowing the EPSPs to coincide later during the sweep. We found evidence for differential delay of excitation as the underlying mechanism in a total of two fast sweep direction selective neurons and one slow sweep direction selective neuron.

Reconstructions could also reproduce FM responses in neurons for which the response to 80 dB SPL tones did not offer an obvious explanation for their FM selectivity. The neuron shown in Figure 9C is one of two neurons in which we could reconstruct the responses to fast FM sweeps, but for which the underlying mechanism was more subtle than in the neurons displayed in Figures 9A,B. As the difference reconstruction gave the best estimation of the FM response, the generation of direction selectivity might be linked to a sequential activation of frequency channels that favors responses to downward modulated sweeps. In six slow sweep direction selective neurons we could reconstruct the FM responses with at least one reconstruction method, suggesting that in these cells the direction selectivity also originated from the spectrotemporal interaction of synaptic inputs.

In other cases, reconstructions were unable to reproduce FM responses. An example is shown in Figure 9D. While we cannot exclude that more elaborate reconstruction methods would fare better, we consider it more likely that this cell received inputs that were already direction selective. Another class of cells in which reconstructions failed consisted of cells in which the response to FM sweeps was dominated by inhibition, but which showed a small EPSP for one direction, leading to a large DSI. Reconstructions did not capture direction selectivity in 6 of 16 fast sweep direction selective neurons and in 16 of 27 slow sweep direction selective neurons.

**Figure 10:**
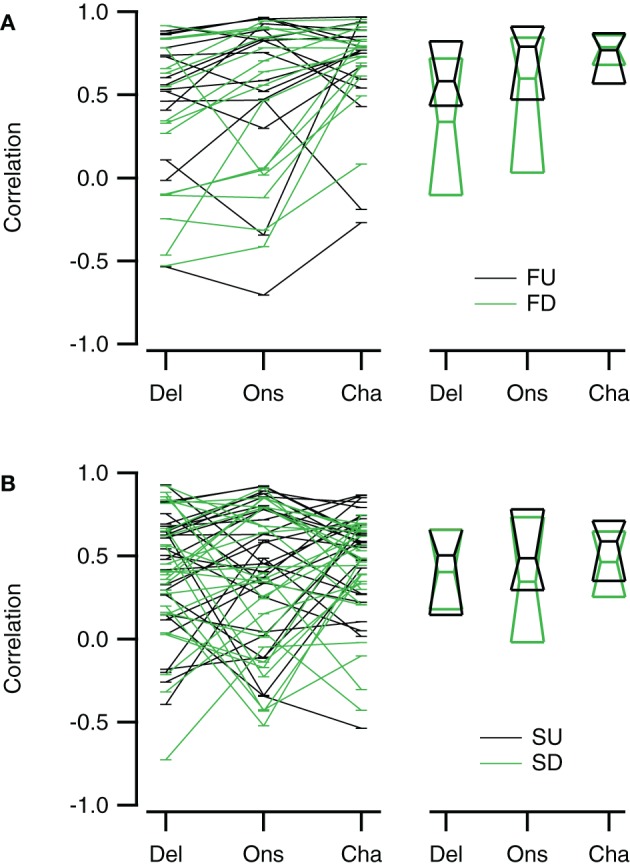
**Reconstructions of direction selective neurons generally correlate well with FM sweep evoked responses. (A)** Correlations of the Delay (“Del”), Onset (“Ons”), and Channel (“Cha”) reconstructions to responses to fast upward (FU, black) and fast downward (FD, green) FM sweeps (*n* = 16). **(B)** Correlation of the three reconstructions to responses evoked by slow upward (SU, black) and slow downward (SD, green) FM sweeps (*n* = 27). Scatter plots with lines connecting reconstructions of the same cell are shown on the left; median values and first and third quartiles are given on the right.

Reconstructions generally resembled the recorded FM evoked responses well. To have a measure for the quality of the reconstruction that is sensitive to the shape and overall timing of the response, but not to its amplitude, we chose to correlate our reconstructions with the recorded responses to FM sweeps. These correlations were limited to a time window matching the duration of the FM stimulus. Figure 10 summarizes the correlations for direction selective neurons (|DSI| >0.33). Comparing the three reconstructions, the correlations were more similar for fast upward (Kruskal–Wallis H = 1.71, *df* = 2, *P* = 0.43) than for fast downward sweeps (Kruskal–Wallis H = 7.48, *df* = 2, *P* < 0.05). The Channel reconstruction yielded the highest correlations values, while the Delay and Onset reconstructions showed slightly lower correlations (Figure 10A; Table 2). The reconstructions of slow sweeps also yielded fairly high correlation values (Figure 10B; Table 2). The correlation values were similar for the three reconstructions of slow upward (Kruskal–Wallis H = 0.95, *df* = 2, *P* = 0.62) and slow downward sweeps (Kruskal–Wallis H = 0.35, *df* = 2, *P* = 0.83). The correlations of Delay, Onset, and Channel reconstructions of slow sweeps were generally lower than for fast sweeps, probably owing to the longer duration (Table 2).

**Table 2:** **Correlations and selectivity errors of the three types of reconstructions**.

		**Direction selectivity**				**Rate selectivity**			
		**Fast sweeps**		**Slow sweeps**		**Up sweeps**		**Down sweeps**	
		**Up**	**Down**	**Up**	**Down**	**Fast**	**Slow**	**Fast**	**Slow**
Delay	Correlation	0.58	0.34	0.50	0.40	0.52	0.64	0.69	0.39
	Error	2.1 mv		2.07 mV		1.26 mV		1.76 mV	
Onset	Correlation	0.79	0.60	0.49	0.35	0.57	0.69	0.59	0.64
	Error	2.4 mV		2.38 mV		2.2 mV		1.9 mV	
Channel	Correlation	0.77	0.79	0.59	0.46	0.88	0.68	0.77	0.54
	Error	1.58 mV		2.24 mV		2.65 mV		1.29 mV	

*Errors and correlations are based on responses to contralateral stimulation at 80 dB SPL*.

**Figure 11:**
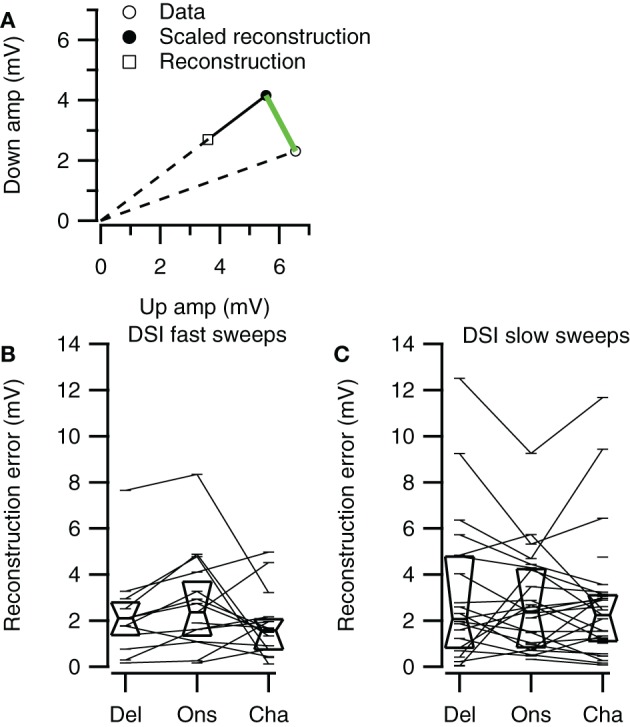
**Direction selectivity errors of the three reconstruction methods. (A)** Illustration of the selectivity error calculation. Along a line from the origin through the reconstructed data point we determined the point with equal distance to the origin as the data point. The distance between this point and the data point (green line) was used as the selectivity error. **(B)** Selectivity errors for fast up- and downward sweeps (Del, *n* = 13; Ons, *n* = 15; Cha, *n* = 16). **(C)** Selectivity errors for slow up- and downward sweeps (Del, *n* = 21; Ons, *n* = 24; Cha, *n* = 27). Box plots show quartiles and medians.

Selective responses to fast sweeps were best reproduced by the Channel reconstruction whereas selective responses to slow sweeps were best reproduced by the Delay reconstruction. While the good correlations indicate that the reconstructions captured the temporal pattern of the response, it does not describe how well the reconstructions yielded the correct relative response amplitude. However, the correlation between the direction or rate selectivity indices of measured data and the reconstructions were poor (data not shown). A main reason for the poor correlation was that the measured EPSP sizes were often small, which had the effect that small errors in the reconstruction amplitudes led to large errors in DSI or RSI. To have a measure for the relative postsynaptic potential amplitude difference that was less sensitive to the absolute size of responses, we calculated the geometrical distance between a point describing the amplitudes of two FM sweeps and a second point with equal distance to the origin that has the same rate or DSI as resulting from the reconstruction amplitudes (Figure 11A). The calculated distance gives a selectivity error in millivolt that is independent of the absolute amplitudes of the reconstructions. For direction selective neurons (|DSI|>0.33) the selectivity error was generally in the range of a few millivolts. The median values for the three types of reconstructions were similar for fast (Kruskal–Wallis H = 0.21, *df* = 2, *P* = 0.90) and for slow sweeps (Kruskal–Wallis H = 1.57, *df* = 2, *P* = 0.46; Figures 11B,C; Table 2).

### Reconstruction of rate selective FM responses from simple tone responses

**Figure 12:**
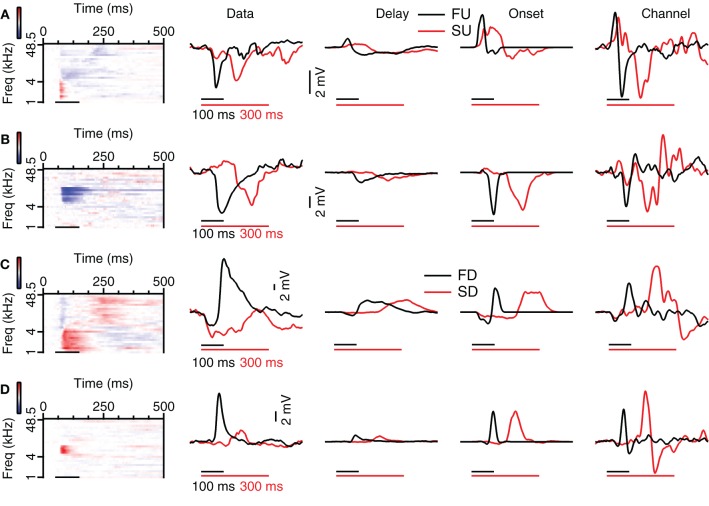
**Reconstruction of fast and slow FM sweeps. (A)** Responses to 80 dB SPL tones (left) of a neuron with a small selectivity error for the Delay and the Onset reconstruction. The average of the truncated membrane potential (Data) is given next to the three different reconstructions (Delay, Onset, Channel). Resting membrane potential was −67 mV. **(B)** Neuron with a large selectivity error for the Delay reconstruction and smaller errors for the Onset and Channel reconstruction. Resting membrane potential was −67 mV. **(C)** Neuron with large selectivity errors for all three reconstruction methods. Resting membrane potential was −70 mV. **(D)** Cell with high reconstruction correlation values, capturing the temporal aspect of the response, but large selectivity error, showing that the reconstruction did not describe the relative amplitudes well. Resting membrane potential was −73 mV. Abbreviations: FU, fast upward; FD, fast downward; SU, slow upward; SD, slow downward.

None of the three reconstruction methods generally reproduced rate selectivity well. Four different examples of reconstructions for two different FM sweep rates are shown in Figure 12. The reconstructions of the rate selective neuron shown in Figure 12A had low correlations for all three types of reconstructions in the time window matching the FM stimulation, among others because the EPSP that was present at low frequencies in the responses to 80 dB SPL tones did not show up in the recorded response to FM sweeps. The Delay, Onset, and Channel reconstructions yielded low selectivity errors (0.02, 0.06, 0.09 mV, respectively), indicating that all reconstructions captured the small relative difference in peak amplitude of the response. However, the small selectivity errors in this case probably result from the very small amplitude of the depolarization during FM stimulation, since the reconstructions showed a poor correlation with the FM responses. A total of 5 out of 7 upward sweep rate selective neurons could also not be reconstructed owing to the small size of the depolarization.

An example of a rate selective cell in which the relative amplitudes of the responses to FM sweeps could be approximated is shown in Figure 12B. Selectivity errors were low (Del: 0.52, Ons: 0.53, Cha: 0.36 mV). In addition, the reconstructions were also well correlated to the FM responses in the time window matching the FM stimulation (all *r* > 0.77). The high correlation values probably resulted from the successful reconstruction of the inhibitory potential, which was correct with respect to timing, and which dominated the responses to 80 dB SPL tones. The EPSP evoked by the slow sweep rate was fairly small but was reproduced in the Delay and the Onset reconstruction. Only in 2 out of 7 upward sweep rate selective neurons the relative amplitudes of the EPSPs could be approximated.

One cause for a failure to reconstruct rate selectivity was the underestimation of inhibitory inputs. The rate selective neuron in Figure 12C showed high correlations between reconstructions and the recorded response (all *r* > 0.65). However, the selectivity errors for Delay, Onset, and Channel reconstruction were all extremely high (12.6, 11.0, 15.9 mV, respectively), because all reconstructions underestimated the inhibition during the slow sweep. In a total of 5 out of 8 downward sweep rate selective neurons the reconstructions reflected the FM responses poorly. Possible reasons for the weak reconstructions could be very small EPSP amplitudes, potential shortcomings of our reconstruction methods or rate selective inputs.

Another reason why reconstructions could fail to reproduce rate selective responses was because they overestimated excitatory inputs. The correlation values during stimulation of the reconstructions in Figure 12D were also high (all *r* > 0.62). The reconstruction selectivity errors were quite large (Del: 4.7, Ons: 5.4, Cha: 7.4 mV) because all reconstructions overestimated the amplitude of the slow downward sweeps. In 3 out of 8 downward sweep rate selective neurons some of the reconstructions yielded larger amplitudes for the preferred sweep rates, but in all these three neurons the reconstructions were fairly poor. Possible reasons for the weak performance of our reconstructions include that rate selectivity was more accidental, imperfections in the reconstruction algorithms, or the possibility that rate selectivity was inherited from upstream nuclei via rate selective inputs.

**Figure 13:**
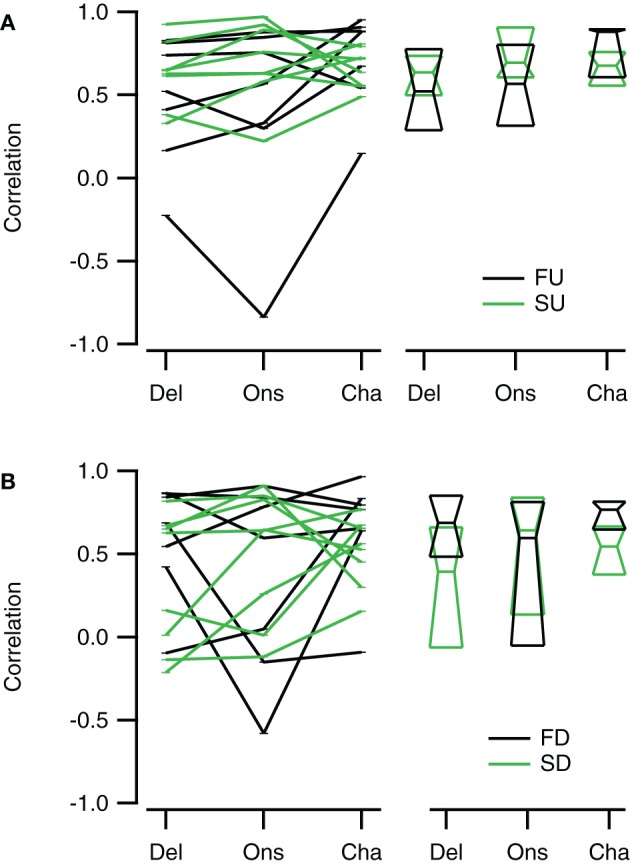
**Reconstructions of rate selective neurons correlate well to FM sweep evoked responses. (A)** Correlations of the Delay (“Del”), Onset (“Ons”), and Channel (“Cha”) reconstructions to responses to fast upward (FU, black) and slow upward (SU, green) FM sweeps (*n* = 8). **(B)** Correlation of the three reconstructions to responses evoked by fast downward (FD, black) and slow downward (SD, green) FM sweeps (*n* = 8). Scatter plots with lines connecting reconstructions of the same cell are shown on the left; median values and quartiles are given on the right.

**Figure 14:**
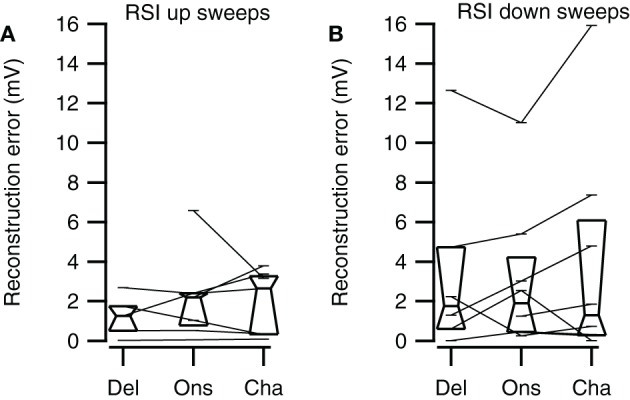
**Rate selectivity errors of the three reconstruction methods. (A)** Selectivity errors for fast and slow upward sweeps (Del, *n* = 5; Ons, *n* = 7; Cha, *n* = 7). **(B)** Selectivity errors for fast and slow downward sweeps (Del, *n* = 6; Ons, *n* = 8; Cha, *n* = 8). Box plots show quartiles and medians.

Channel reconstructions yielded the highest correlation values for fast sweeps and onset reconstructions resulted in the highest correlation values for slow sweeps in rate selective neurons (RSI >0.6875; Figure 13). Although the Delay, Onset, and Channel reconstructions generally yielded high correlations for up- and downward reconstructions, there were a larger number of downward reconstructions with relatively low correlation values, resulting in a larger spread. The three reconstruction methods yielded similar correlations for fast upward (Kruskal–Wallis H = 3.27, *df* = 2, *P* = 0.21), slow upward (Kruskal–Wallis H = 0.65, *df* = 2, *P* = 0.70), fast downward (Kruskal–Wallis H = 0.72, *df* = 2, *P* = 0.68), and slow downward sweeps (Kruskal–Wallis H = 1.34, *df* = 2, *P* = 0.51). The Channel reconstructions resulted in the highest correlation values for fast sweeps while the Onset reconstruction gave the highest correlation values for slow sweeps (Table 2). Finally, we also calculated the selectivity error for rate selective neurons, which had quite variable median values for the Delay, Onset, and Channel reconstruction (Figure 14; Table 2). The three reconstructions yielded similar reconstruction errors for upward sweeps (Kruskal–Wallis H = 2.89, *df* = 2, *P* = 0.24) but different errors for downward sweeps (Kruskal–Wallis H = 17.38, *df* = 2, *P* < 0.001).

## Discussion

Our results show that neurons in the dorsal cortex of the mouse inferior colliculus can respond selectively to the direction or rate of FM sweeps. We observed direction and rate selectivity both in suprathreshold spiking and in subthreshold EPSPs. Action potential based selectivity was predicted well by membrane potential based selectivity in case of direction but poorly in the case of rate. Neurons with similar FRAs also had similar responses to FM sweeps, suggesting that responses to FM sweeps could be predicted from FRAs. Our reconstructions show that FM evoked responses could be reconstructed to a large extent from responses to simple tones, suggesting that some of the neurons in the dorsal cortex of the inferior colliculus can generate FM direction and rate selective responses.

### Direction selectivity in the dorsal inferior colliculus

Based on both suprathreshold and subthreshold responses, we found FM direction selective neurons in the dorsal cortex of the mouse inferior colliculus. We found that the percentage of direction selective cells in the mouse dorsal cortex was relatively low, with values ranging between 13 and 24% for contralateral stimulation. This is still within the range of values observed in the rat central nucleus, where estimates from about 10% to over 50% of cells have been obtained (Poon et al., 1991, 1992; Felsheim and Ostwald, 1996; Kuo and Wu, 2012). As the inferior colliculus responds not only to contralateral but also to ipsilateral stimulation, we also determined the number of direction selective neurons in response to FM sweeps presented to the ipsilateral ear. With values ranging between 10 and 13%, fewer neurons were direction selective in response to FM sweeps presented to the ipsilateral ear. The DSI distribution indicated no clear preference for up- or downward sweeps in the dorsal cortex; only the EPSP-based DSIs for ipsilateral stimulation were slightly shifted toward higher (upward) values. In rats the proportion of up- and downward direction selective neurons in the inferior colliculus are typically similar, while in bats relatively many downward selective neurons are found, in accordance with the structure of their calls (Clopton and Winfield, 1974; Fuzessery, 1994; Felsheim and Ostwald, 1996; Andoni et al., 2007; Andoni and Pollak, 2011).

### Rate selectivity in the dorsal inferior colliculus

A subset of neurons in the dorsal cortex of the inferior colliculus responded selectively to the rate of FM sweeps. Large RSIs were common for suprathreshold responses but fairly rare for subthreshold responses. A high percentage of rate selective neurons has been observed in extracellular studies of the central nucleus of the inferior colliculus, while only 6–21% of cells in our dataset were selective for modulation rate (Felsheim and Ostwald, 1996; Fuzessery et al., 2006). A direct comparison is however difficult because of differences in the presented stimuli, including the number of different rates presented, the intensity of the stimuli, and the type of FM sweeps (logarithmic vs. linear). Both the analysis of action potentials and EPSPs revealed fewer modulation rate selective neurons if sweeps were presented to the ipsilateral ear than to the contralateral ear. However, these neurons showed higher rate selectivity than cells that were rate selective for contralateral stimulation. The functional significance of these differences between ipsi- and contralateral stimulation is not yet clear.

### Spike threshold effects on FM selectivity

FM selectivity indices for direction and rate were generally larger when based on action potentials compared to EPSPs. This observation can be attributed to a sharpening effect of the spike threshold on selectivity. Small changes in membrane potential can result in large changes in action potential firing, leading to larger action potential based selectivity indices. We previously found a clear effect of the non-linear relation between membrane potential and spike probability on tuning for amplitude modulated (AM) responses in the central nucleus of the inferior colliculus (Geis and Borst, 2009). A spike threshold effect on FM selectivity has been put forward in studies of the direction and rate selectivity in the central nucleus of the inferior colliculus and the auditory cortex (Gittelman et al., 2009; Ye et al., 2010; Gittelman and Li, 2011). Many direction- or rate-selective neurons in our dataset only fired action potentials when stimulated with the preferred sweep. In addition, neurons with high selectivity indices often fired only few action potentials. In these cases there was often little underlying selectivity in EPSP sizes, suggesting that the rate selectivity for action potentials in these cells may have been accidental. Both points support the notion that spike threshold effects increase FM selectivity in the dorsal cortex of the inferior colliculus as well.

### Generation of FM selectivity in the dorsal inferior colliculus

In our study, we compared the response to simple tones with the response to FM sweeps. This step can provide information about the question to what extent the FM responses are generated *de novo* by the recorded cell, or result from synaptic integration in auditory stations that are located upstream from the dorsal cortex. One indication that FM responses can be locally generated was our observation that pairs of neurons with similar responses to FM sweeps also had similar FRAs. This does not constitute proof for a causal relation since neurons can inherit both FM responses and tone responses, but did provide an impetus to test whether responses to FM sweeps can be predicted from the responses to simple tones composing the FRA.

Further evidence for the local generation of FM sweeps was obtained by comparing the FRAs for direction selective cells with the results obtained in the literature. Two main mechanisms have been proposed for the generation of direction selective responses (reviewed in Fuzessery et al., 2011; Pollak et al., 2011). The first mechanism relies on asymmetrical inhibition, resulting in direction-dependent differences in the relative timing of excitatory and inhibitory inputs. In about half of the FM direction selective neurons in our sample this mechanism most likely was responsible for the direction selectivity. In these direction selective cells the inhibitory area was at high frequencies for the upward selective cells and at low frequencies for downward direction selective cells. As a result, in the preferred direction, excitation would clearly precede the inhibition, whereas in the other direction, excitation and inhibition would coincide. In most of these cells, further support was obtained by the reconstructions, which showed that this mechanism provided a sufficient explanation for the observed direction selectivity.

The second proposed mechanism concerns the timing of excitatory inputs. In some neurons we recorded, differential latencies and EPSP time course appeared to underlie the response selectivity, including some direction selective neurons that displayed only excitatory FRAs. Even though this mechanism has been suggested to be responsible for creating direction selectivity in many studies (e.g., Suga and Schlegel, 1973; Phillips et al., 1985; Fuzessery et al., 2011), evidence for this mechanism is not as convincing as for the asymmetric inhibition. By reproducing the direction selectivity in the reconstructions we could show that this mechanism was a sufficient explanation for the creation of direction selectivity in some neurons in the dorsal cortex.

The presence of multiple mechanisms differs from the rat central nucleus, where it was found that asymmetric inhibition was responsible for direction selectivity in all neurons that were direction selective (Kuo and Wu, 2012), but agrees with results in the auditory cortex (Razak and Fuzessery, 2008; Ye et al., 2010) and in the bat central nucleus of the inferior colliculus (Fuzessery et al., 2011), where asymmetric inhibition, differential delay, and upstream mechanisms all are thought to contribute, even though it is still partly unclear to what extent these mechanisms are inherited from upstream nuclei.

The mechanisms underlying the observed rate selectivity were harder to deduce. The generation of rate selective responses is also thought to rely on the spectrotemporal interaction of synaptic inputs (reviewed in Fuzessery et al., 2011; Pollak et al., 2011). The mechanisms underlying rate selectivity have been mostly studied in the bat central nucleus of the inferior colliculus, where neurons generally show narrow excitatory tuning curves, in contrast to the broad tuning curves observed in our study. As the majority of rate selective neurons in our sample showed only small EPSPs in response to stimulation with FM sweeps, and the more responsive rate selective cells were poorly reconstructed, it remains to be established that clear rate selectivity can be generated within the dorsal cortex of the inferior colliculus.

### Comparison of the three reconstruction methods

To explore which aspect of the FRAs contributed to FM evoked response, we reconstructed FM evoked responses from responses to simple tones with three methods, each emphasizing a different response characteristic. Without employing any fitting, the reconstructions could reproduce responses to FM sweeps in the majority of neurons that showed direction selectivity to fast FM sweeps, but also in other cells. This is also remarkable because the three methods did not model the activation of voltage-dependent ion channels. In the cases where reconstruction was unsuccessful, our method does not allow to discriminate whether non-linear integration within the recorded neuron was responsible for the mismatch, or whether FM responses were inherited, although in cases where the fit was very poor the latter seems more likely.

The simplest reconstruction method we employed only considered the time shift introduced by the FM sweep. A comparable method has been utilized to reconstruct FM evoked responses in the auditory cortex (Ye et al., 2010). This Delay reconstruction often did not capture the temporal fine structure of FM evoked response and therefore correlations were mostly lower than for the other types of reconstructions. Although other reconstructions could yield lower selectivity errors, the Delay reconstruction resulted in the most consistent selectivity error values, suggesting that this simple method is the most stable one. Focusing on the response onset, the Onset reconstruction could reproduce the temporal pattern in response to fast sweeps better than in response to slow sweeps. The selectivity errors of the onset reconstruction usually exceeded that of the other methods, emphasizing the lack of temporal integration as a drawback of this reconstruction method. The Channel reconstruction yielded the highest correlations and the lowest selectivity errors for fast sweeps, suggesting that for fast FM sweeps the concept of different frequency channels that are sequentially activated provides a good approximation. In reproducing responses to slow FM sweeps, the Channel reconstruction performed on a level comparable to the Delay reconstruction. As responses to simple tones do not contain information about direction or rate selectivity in response to FM sweeps, successful reconstructions of direction or rate selective responses indicate generation of this selectivity in neurons of the dorsal cortex of the inferior colliculus.

### Limitations of the present reconstruction methods

Even though we did not measure the underlying synaptic conductances and even though only a single duration was used for the simple tone stimuli, these data already allowed us to reconstruct the FM response with high fidelity in many cases. Obviously, it should be possible to obtain even more accurate reconstructions if a more extensive set of simple tone responses were obtained. Voltage clamp studies at different holding potentials can generate more quantitative information about both excitatory and inhibitory conductances than the current clamp recordings we made, although the high series resistance typically obtained for *in vivo* whole-cell recordings and the spatial clamp problems that are always present in intact neurons make appropriate controls essential (Kuo and Wu, 2012). We now simply added inhibitory and excitatory potentials, without taking into account the much smaller driving force for the IPSPs. The exact driving force was unknown in our experiments, because of the large access resistance of electrodes and the resulting incomplete dialysis of the cell with internal medium. Stimuli at different duration can provide information about the onset and offset of these synaptic conductances, which can be helpful to better match the tone duration to the time spent at each frequency at the different sweep rates, to take into account the effect of adaptation to prolonged tones, and to better include the responses to tone offsets. In combination with information about passive properties and voltage-dependent ion channels, an even better prediction of intracellular FM responses should thus be possible.

Another aspect of the quantitative relation between simple tones and FM sweeps that can still be further improved is related to the question what the contribution of individual inputs is to the measured FRA. As the FM sweep traverses this area, it will activate different inputs (“frequency channels”), depending on the individual receptive fields of the different presynaptic neurons. In our third model, we assumed that if simple tone responses changed little for adjacent stimulus frequencies, they were originating from the same set of presynaptic inputs. To test this more directly, a possible approach could be a systematic test for stimulus-specific adaptation using so-called “oddball” paradigms (Malmierca et al., 2009). If responses to simple tones of different frequencies co-adapt, it can be assumed that they are mediated by the same set of inputs (frequency channel). In combination with the conductance measurements, such an approach would also make it easier to discriminate between the appearance of inhibitory conductance and the disappearance of an excitatory conductance, which was now one of the hardest decisions in our current Channel model approach. Thus, it can be expected that an even better match between simple tone responses and FM responses can be obtained than was currently possible.

### Conflict of interest statement

The authors declare that the research was conducted in the absence of any commercial or financial relationships that could be construed as a potential conflict of interest.
